# Case Report: Three Rare Cases of Ectopic ACTH Syndrome Caused by Adrenal Medullary Hyperplasia

**DOI:** 10.3389/fendo.2021.687809

**Published:** 2021-07-01

**Authors:** Yu Cheng, Jie Li, Jingtao Dou, Jianming Ba, Jin Du, Saichun Zhang, Yiming Mu, Zhaohui Lv, Weijun Gu

**Affiliations:** ^1^ Department of Endocrinology, Chinese PLA General Hospital, Beijing, China; ^2^ Department of Pathology, Chinese PLA General Hospital, Beijing, China

**Keywords:** ACTH-dependent Cushing’s syndrome, ectopic ACTH syndrome, adrenal medullary hyperplasia, immunohistochemical staining, bilateral adrenalectomy

## Abstract

Ectopic ACTH syndrome (EAS) accounts for 10–20% of endogenous Cushing’s syndrome (CS). Hardly any cases of adrenal medullary hyperplasia have been reported to ectopically secrete adrenocorticotropic hormone (ACTH). Here we describe a series of three patients with hypercortisolism secondary to ectopic production of ACTH from adrenal medulla. Cushingoid features were absent in case 1 but evident in the other two cases. Marked hypokalemia was found in all three patients, but hyperglycemia and osteoporosis were present only in case 2. All three patients showed significantly elevated serum cortisol and 24-h urinary cortisol levels. The ACTH levels ranged from 19.8 to 103.0pmol/L, favoring ACTH-dependent Cushing’s syndrome. Results of bilateral inferior petrosal sinus sampling (BIPSS) for case 1 and case 3 confirmed ectopic origin of ACTH. The extremely high level of ACTH and failure to suppress cortisol with high dose dexamethasone suppression test (HDDST) suggested EAS for patient 2. However, image studies failed to identify the source of ACTH secretion. Bilateral adrenalectomy was performed for rapid control of hypercortisolism. After surgery, cushingoid features gradually disappeared for case 2 and case 3. Blood pressure, blood glucose and potassium levels returned to normal ranges without medication for case 2. The level of serum potassium also normalized without any supplementation for case 1 and case 3. The ACTH levels of all three patients significantly decreased 3-6 months after surgery. Histopathology revealed bilateral adrenal medullary hyperplasia and immunostaining showed positive ACTH staining located in adrenal medulla cells. In summary, our case series reveals the adrenal medulla to be a site of ectopic ACTH secretion. Adrenal medulla-originated EAS makes the differential diagnosis of ACTH-dependent Cushing’s syndrome much more difficult. Control of the hypercortisolism is mandatory for such patients.

## Introduction

Adrenocorticotropic hormone (ACTH)-dependent Cushing’s syndrome can be classified as either Cushing’s disease or ectopic ACTH syndrome (EAS). It is estimated that EAS is responsible for 10–20% of all cases of Cushing’s syndrome. The frequencies of tumors associated with EAS vary among series (1). In the 1970s, EAS were mainly represented by small-cell lung carcinomas (SCLCs). But the most prevalent tumor of EAS has recently shifted from SCLCs to neuroendocrine tumors (NETs), and particularly bronchial carcinoids in the recent literature (2–4). Other origins of tumors responsible for EAS are the thymus (5), pancreas, and thyroid. Pheochromocytoma also has been reported to have ectopic ACTH secretion and accounts for approximately 5% of EAS cases (6). Adrenal medullary hyperplasia, characterized by an increased medullary cell mass, is even more rare for excess catecholamine production (7, 8). Hardly any case of adrenal medullary hyperplasia was previously reported to ectopically secrete ACTH according to our systematic search of publications. Here, we reported three cases of EAS associated with adrenal medullary hyperplasia in our center.

## Case Description

Case series results are summarized in **Tables 1** and **2** and are individually described here.

**Table 1:** Summary of Characteristics and Laboratory Findings at Presentation.

	Case 1	Case 2	Case 3	Normal Range
Age at diagnosis	23y	28y	31y	
Sex	Female	Male	Male	
BMI	20.8	28.0	31.6	<28kg/m^2^
HbA1c	5.7	5.6	5.4	<6.5%
Glucose after 75g OGTT				
0min	3.9	8.65	4.8	3.4-6.1mmol/L
60min	6.95	16.48	8.34	<11.1mmol/L
120min	7.06	17.12	7.26	≥7.8mmol/L
Blood pressure	130/80	150/100	130/90	<140/90mmHg
Z score	-1.3	-4.0	0.2	>-1
L1-L4 spine				
Serum potassium	2.56	1.29	2.66	3.5-4.5mmol/L
Serum cortisol				
0AM	1007.19	3048.0	785.28	0-165.7nmol/L
8AM	1229.25	3577.9	1080.58	198.7-797.5nmol/L
4PM	1152.32	3394.7	164.21	85.3-459.6nmol/L
Urine free cortisol	6674.8	47862	6358.8	98.0-500.1nmol/24h
Serum ACTH				
0AM	13.3	88.8	38.4	
8AM	19.8	103.0	42.0	<10.12pmol/L
4PM	23.5	62.6	66.4	
**LDDST**				
Serum cortisol	1997.19		850.21	<50nmol/L
Serum ACTH	24.4	_	28.4	
Urine free cortisol	7499.5		6419.9	<98.0nmol/24h
**HDDST**				
Serum cortisol	1673.45 (136%)	3789.9 (106%)	1793.09 (166%)	<50%
Serum ACTH	22.6	100.0	15.7	
Urine free cortisol	4243.3 (63%)	50800 (106%)	5697.0 (90%)	<50%
**DDAVP**				
ACTH max/ACTH basal	7.11	_	5.77	
**BIPSS**				
Central/peripheral ACTH at basal	0.93	_	1.02	
Central/peripheral ACTH after DDAVP stimulation	1.5	_	0.67	

**Table 2:** Assessment Before and After Bilateral Adrenalectomy.

	Case 1	Case 2	Case 3
Serum ACTH (pmol/L)	Before 3m 12m	Before 3m 12m	Before 3m 12m
0AM	13.3 1.77 -	88.8 1.1 1.1	38.4 3.53 7.3
8AM	19.8 11.9 -	103.0 4.06 54.2	42.0 7.33 86.1
4PM	23.5 10.1 -	62.6 4.00 12.6	66.4 6.64 49.3
Serum Cortisol (nmol/L)	Before 3m 12m	Before 3m 12m	Before 3m 12m
0AM	1007.19 197.34 -	3048.0 134.65 40.50	785.28 121.81 <25.7
8AM	1229.25 304.11 -	3577.9 <25.7 31.37	1080.58 342.89 231.36
4PM	1152.32 123.76 -	3394.7 130.54 85.81	164.21 <25.7 <25.7

### Case 1

**Figure 1:**
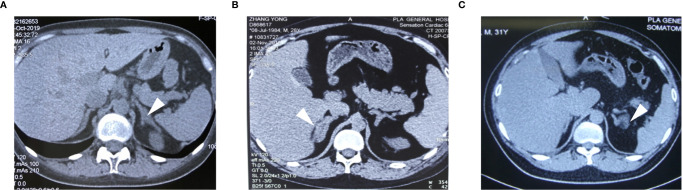
The CT images of the adrenal glands for case1 **(A)**, case 2 **(B)** and case 3 **(C)**. Adrenal contrast-enhanced CT showed adrenal hyperplasia (arrows).

**Figure 2:**
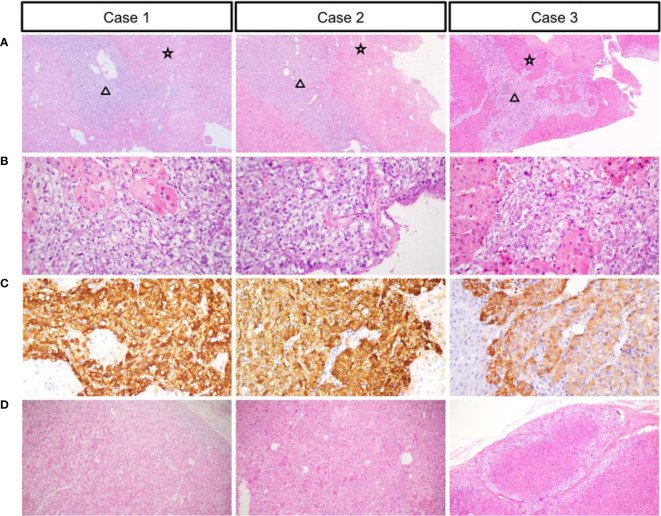
Low power views showing the thickened medulla (black triangle) and hyperplastic adrenal cortex (black star) **(A)**. High-power views of the medulla **(B)**. Chromogranin expression in the medullary cells **(C)**. High-power views of the cortex **(D)**.

**Figure 3:**
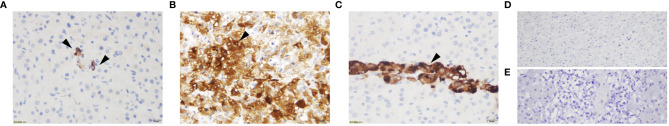
ACTH immunostaining of the adrenal glands for case1 **(A)**, case 2 **(B)**, case 3 **(C)**, adrenal cortex of normal control **(D)** and adrenal medulla of normal control **(E)**. ACTH staining showed sporadic positive ACTH staining for case 1, numerous adrenal medulla cells positive for ACTH for case 2, focal positive ACTH staining for case 3 and negative ACTH staining for normal control.

A 23-year-old woman was admitted to our department presented with a one-month history of general fatigue, acne and hirsutism. Blood pressure was 130/80 mmHg. Her weight was 55 kg, and height was 162.5 cm (Body mass index 20.8Kg/m^2^). She had no typical symptoms of Cushing’s syndrome such as moon face, central obesity, supraclavicular fat accumulation, or buffalo hump. Stria was also invisible. Hypokalemia was evident, which could not be normalized with oral and intravenous potassium supplementation until spironolactone (60mg tid) was added. The level of HbA1c was 5.4%, and blood glucose levels were within normal range after a 75-g oral glucose tolerance test (OGTT). Dual-energy X-ray absorptiometry (DXA) bone densitometry suggested osteopenia with a Z-score of -1.3 at L1-L4 spine. Biochemical workup (**Table 1**) revealed loss of circadian rhythm, elevated 24-h urinary cortisol levels and failure to suppress cortisol with classical low dose dexamethasone suppression test (LDDST). Plasma ACTH level was elevated to 19.8 pmol/L, favoring ACTH-dependent Cushing’s syndrome. Desmopressin stimulation resulted in ACTH increase by about 700%. But cortisol was not suppressed after high dose dexamethasone intake. Magnetic resonance imaging (MRI) showed normal pituitary gland (**Supplementary Figure 1A**). So next, bilateral inferior petrosal sinus sampling (BIPSS) was performed and showed a central-to-peripheral ACTH gradient < 2 at baseline and < 3 after desmopressin stimulation. Finally, diagnosis of EAS was considered. The lesion was not localized by CT scans. Further evaluation with ^68^Ga-DOTATATE PET/CT and 18F-PET/CT still failed to identify the source of ACTH secretion. Only bilateral adrenal hyperplasia was observed *via* image studies (**Figure 1A**). As steroidogenesis inhibitors are unavailable in China, mifepristone can aggravate hypokalemia, and the cortisol level remained unchangeable after short-acting octreotide injection, bilateral adrenalectomy was performed for rapid control of hypercortisolism. Transperitoneal approach was used with the patient positioned in left and right lateral positions for right and left glands, respectively. A meticulous surgical technique was used to best expose the adrenal veins to clip them before division followed by excising the glands. The patient had minimal blood pressure variations during surgery. After surgery, a 20mg dose of hydrocortisone per day was given. The level of serum potassium normalized without any supplementation. The level of serum sodium was around 140mmol/L. Blood pressure maintained between 100-110/60-70mmHg. ACTH level significantly decreased at the latest follow-up (3 months after surgery, **Table 2**), indicating adrenal glands to be the origin of excess ACTH. Consistently, the low power view of histopathological image showed a thickened adrenal medulla, more than a third of the total adrenal thickness in both glands, which met the criteria of adrenal medulla hyperplasia in WHO 2017 classification of endocrine tumors (**Figure 2A**). The high power view showed diffuse hyperplasia of the medullary cells, and the medullary cells showed size and shape variability in areas of diffuse hyperplasia (**Figure 2B**). The medullary cells stained positive for chromogranin A (**Figure 2C**). Histopathology revealed bilateral diffuse cortex hyperplasia (**Figure 2D**). Immunostaining showed sporadic positive ACTH staining (**Figure 3A**) located in adrenal medulla cells. To rule out the possibility of non-specific staining, ACTH staining was simultaneously performed in adrenal tissues from a patient undergoing adrenalectomy due to renal cell carcinoma, and no positive cells were spotted neither in adrenal cortex nor adrenal medulla (**Figures 3D, E**).

### Case 2

A 28-year-old man complained with a six-month history of acne and three-month history of moon face, general fatigue and hypertension. Physical examination revealed a blood pressure of 150/90 mmHg, weight of 78 kg, and height of 167 cm (BMI 27.9 Kg/m^2^). He displayed overt cushingoid features with central obesity, dorsocervical and supraclavicular fat pads, abdominal striae, skin bruises, acne, and facial plethora. Laboratory examination showed severe hypokalemia (1.2mmol/L). A 75-g OGTT confirmed diabetes mellitus with a fasting blood glucose level of 8.65 mmol/l and a 2-h glucose level of 17.12 mmol/L, although HbA1c level was only 5.6%. DXA showed severe osteoporosis with a Z-score of -4.0 at the L1-L4 spine. Endocrinological investigation (**Table 1**) identified severe hypercortisolism with loss of circadian rhythm and elevated 24-h urinary cortisol levels. Plasma ACTH level was significantly elevated to 103 pmol/L, confirming ACTH-dependent Cushing’s syndrome. The serum cortisol level was not suppressed after a high dose dexamethasone suppression test (HDDST). MRI revealed no pituitary adenoma (**Supplementary Figure 1B**). We offered BIPSS for ACTH assays to identify the origin of ACTH, but the patient refused. Nonetheless, EAS was considered given the ACTH level, results of HDDST and pituitary MRI. No lesion was detected in thoracic CT scan. Abdominal CT showed hyperplasia of the right adrenal gland (**Figure 1B**). Pituitary MRI showed a mass in the left nasal cavity and left ethmoidal sinus (**Supplementary Figure 2**), and PET-CT confirmed intense metabolic activity of the mass. The mass was thereafter biopsied. Pathology showed that the mass was composed of small round blue cells with a high nuclear-to-cytoplasmic ratio, rare nucleoli and a nuclear chromatin pattern typical of neuroendocrine-like tumors. The diagnosis of olfactory neuroblastoma was supported by immunohistochemical staining for multiple neuro-endocrine markers, such as CD56, synaptophysin, chromogranin A and S-100. Previously, more than 20 cases of olfactory neuroblastoma presenting with EAS have been reported (9, 10). However, biopsy did not show positive ACTH staining. Surgical removal of the tumor was delayed due to poor general condition of the patient. Upon admission, ketoconazole was still available. At a dose of 800mg/d, the serum cortisol level dropped from 3577.9 to 1159.3nmol/L, but liver damage was already induced with ALT level reaching 300U/L. Therefore, ketoconazole was discontinued and bilateral adrenalectomy was next performed. Similarly, no sudden rise of blood pressure was observed during surgery. After surgery, cushingoid features gradually disappeared. Blood pressure, blood glucose and potassium levels returned to normal ranges without medication. He needed a 7.5-mg dose of prednisone acetate per day. At 3 month after surgery, the ACTH level decreased to 4.06 pmol/L. As the general condition significantly improved, the patient next underwent transnasal endoscopic resection of the tumour mass. ACTH and CRH staining were both negative for the tumor, whereas histopathology showed an increased adrenal medullary cell mass and diffuse hyperplasia of the medullary cells in both adrenal glands (**Figures 2A, B**). The medullary cells stained positive for chromogranin A (**Figure 2C**). Histopathology also revealed bilateral diffuse cortex hyperplasia (**Figure 2D**). Immunohistochemical staining showed numerous adrenal medulla cells positive for ACTH (**Figure 3B**). A year after bilateral adrenalectomy, the patient was admitted for regular follow-up. The 8AM ACTH markedly elevated to 54.2pmol/L but was suppressed below 1.11pmol/L after 1mg dexamethasone administration, suggesting that the up-regulated ACTH level was a result of adrenocortical insufficiency. The patient is currently under regular follow-up and remains well for 7 years.

### Case 3

A 31-year-old man presented with a two-month history of general fatigue, acne, moon face and hyperpigmentation. Physical examination revealed a blood pressure of 130/90 mmHg, weight of 99 kg, and height of 177 cm (BMI 31.6 Kg/m^2^). He showed cushingoid features including moon face, supraclavicular fat accumulation, buffalo hump, facial and truncal acne, ecchymoses and striae. Laboratory examination showed hypokalemia. The level of HbA1c was 5.4%. A 75-g OGTT showed normal glucose homeostasis. Z score at L1-L4 spine was 0.2. The serum levels of ACTH, cortisol, and 24-h urine free cortisol were markedly elevated (**Table 1**). Results of LDDST showed a cortisol value of 840.21 nmol/L after the dexamethasone administration. There was no suppression after HDDST. MRI of the pituitary gland did not showed signs of pituitary adenoma (**Supplementary Figure 1C**). Just like case 1, ACTH max/ACTH basal was as high as 5.77 after desmopressin stimulation. BIPSS showed that there was no evidence of a central-to-peripheral gradient of ACTH at baseline or after desmopressin stimulation. According to these findings, the patient was diagnosed with EAS. Thoracic CT scan was normal. Abdominal CT only showed nodular hyperplasia of the left adrenal gland (**Figure 1C**). The 18F-PET/CT showed increased uptake in both adrenal glands. Octreotide scanning also revealed negative results. As steroidogenesis inhibitors were unavailable, bilateral adrenalectomy was then performed *via* the same approach as case 1 without arterial variations. After surgery, a 20mg dose of hydrocortisone per day was added. Cushingoid features gradually disappeared. The levels of serum potassium remained normal without potassium supplement. Similarly, immunohistochemical staining showed focal positive ACTH staining in hyperplastic adrenal medulla cells (**Figures 2A–C**, **3C**) and nodular hyperplasia of adrenal cortex in both adrenal glands (**Figure 2D**). ACTH level significantly decreased 3 months after surgery (**Table 2**). At the latest follow-up, a year after surgery, the patient complained about hyperpigmentation. Laboratory tests showed that 8AM ACTH level relapsed to 86.1 pmol/L. After LDDST, ACTH level significantly decreased to 18.4 pmol/L. ^68^Ga-DOTATATE PET/CT and pituitary MRI still showed no signs for lesions. All the data indicated that increase of ACTH was the result of cortisol hypofunction. In order to suppress ACTH secretion, we added dexamethasone 0.1875mg at 10PM.

## Discussion

Our case series suggest that severe Cushing’s syndrome can be induced by ectopic production of ACTH caused by adrenal medulla hyperplasia, supported by a drastic decrease of ACTH level after bilateral adrenalectomy, positive ACTH staining in adrenal medulla cells, and the number of ACTH positive cells in proportion to serum ACTH level.

Back in 1980s, researchers showed the presence of immunoreactive ACTH and corticotropin-releasing hormone (CRH) in adrenals (combined cortex and medulla) by radioimmunity assay, immunoaffinity chromatography, and gel filtration chromatography (11). Interestingly, in patients with proven deficiency of pituitary ACTH, administration of 100ug human CRH induced an increase in plasma ACTH level, indicating extra-pituitary source of ACTH (12). Animal experiments revealed the role of the adrenal medulla as the production site of ACTH after CRH stimulation (13). Subsequently, it was demonstrated that human adrenal medulla expressed CRH receptors (14). Strong evidence supports a local, intra-adrenal CRH/ACTH system (15). Adrenal medullary cells possess the potential to secret ACTH into plasma. So it would be reasonable to infer that in our series, the adrenal medulla was the source of ectopic ACTH secretion.

Compared to pheochromocytomas, adrenal medullary hyperplasia is less common. Only case reports or small case series about adrenal medullary hyperplasia have been reported (16, 17), in which patients often represented with a medical history of symptoms and signs of excessive catecholamine excretion, slightly elevated level of catecholamine, and increased adrenal medullary tissue relative to the cortex. However, in our case series, blood pressure only slightly elevated for case 2, most probably related with hypercortisolism. Given the prominent Cushing’s syndrome in our series, the levels of catecholamine was not detected in any of the three cases. But absence of paroxysmal symptoms and blood pressure variations during the adrenal surgery made the possibility of catecholamine excess relatively low. We hypothesized that the potential of ACTH secretion might be limited to a certain subtype of adrenal medulla cells, and hyperplasia of such cells mainly resulted in hypercortisolism rather than excess catecholamine production. The underlying mechanism needs further exploration.

Occult EAS is not rare. A lack of tumor identification has been documented from 12 to 36.5% in different series (1). In some cases, a non-metastatic well-differentiated and indolent neuroendocrine tumor can be discovered after several months or years of follow-up (4). However, some cases remain truly occult. Our findings provide reasonable explanations for, if not all, parts of these truly occult cases. The adrenal medulla has rarely been identified to be the ectopic source of ACTH before, and its incidence might have been underestimated. There might be two reasons. First, in cases with occult EAS, pharmacological treatments of hypercortisolism are fist-line choices in many countries (18), which would not affect the ACTH level. Without removal of adrenal glands, there would be no evidence to suggest adrenal medulla hyperplasia. Secondly, in cases with bilateral adrenalectomy to control hypercortisolism, the ACTH would rapidly relapse to a high level due to adrenocortical hypofunction. If in the short time-window when ACTH level dropped, the patients did not have the ACTH level checked, the clue would also have been missed.

Differential diagnosis between Cushing’s disease and occult EAS can be challenging. Typically, in EAS, the degree of ACTH hypersecretion is much higher compared to Cushing’s disease. CRH or desmopressin stimulation usually results in increase in ACTH and cortisol levels in Cushing’s disease, but not in EAS. The cortisol level is not suppressed with HDDST. However, these tests may not be completely reliable (19). A review concluded that the sensitivity of desmopressin stimulation in the differential diagnosis of ACTH-dependent Cushing’s syndrome was 83% and the specificity was only 62% (20). In certain researches, EAS patients even responded to desmopressin test in about 30-40% of cases (21). So if ACTH-dependent Cushing’s syndrome were biochemically supported and a pituitary adenoma was detected on MRI, the diagnosis of Cushing’s disease would have been arbitrarily made. However, given that pituitary adenoma can be detected in 5~20% normal people (22), we might have preformed pituitary resection to a patient with occult EAS and concomitant non-functional pituitary adenoma. So it must be born in mind that occult EAS is not rare (23). Under such circumstances, BIPSS is very important. BIPSS combined with CRH and/or desmopressin administration is considered the gold standard for distinguishing EAS from Cushing’s disease (24). Absence of a central to peripheral ACTH gradient confirms an ectopic source of ACTH secretion. EAS associated with adrenal medulla hyperplasia might raise another diagnostic problem as in case 2, when a tumor, which has been reported to be associated with EAS, is indeed identified. It is very likely that surgical excision of the tumor is the first-line choice as surgery offers a good chance for cure while maintains adrenal function. With no doubt, hypercortisolism would persist afterwards.

Desmopressin is a long-acting vasopressin analog with selective V2 agonist activity (25). Pituitary ACTH-producing adenoma expresses V2 receptors (V2R). As a result, desmopressin administration produces a significant rise of ACTH secretion in the majority of patients with Cushing’s disease whereas patients with EAS were unresponsive (26). Since CRH is unavailable in China, desmopressin stimulation test instead is applied to distinguishing EAS from Cushing’s disease. Taken the ACTH max/ACTH basal ratio of 1. 5 as a cut point, a significant overlap of the ACTH response to the desmopressin test was found between patients with Cushing’s disease and EAS. But the ratio rarely exceeds 3 in EAS (20). However, we notice that after desmopressin stimulation, ACTH increment was up to 711% for case 1 and 577% for case 3. It has been reported that the adrenal medulla, from many species, exhibits V1 vasopressin receptors, and *via* V1 receptors, arginine-vasopressin stimulates intramedullary the CRH-ACTH system (27). So we hypothesize that the adrenal medulla also exhibit V2R and desmopressin stimulates the intramedullary ACTH production, making distinguishing adrenal medulla-originated EAS from Cushing’s disease more difficult.

In our study, age of three patients at diagnosis was relatively younger than that in previously published series regarding EAS (2–4, 23, 28, 29). The medical history was short, and most common signs were general fatigue and acne. Cushingoid features were absent in case 1 but evident in the other two cases. With respect to cortisol-induced comorbidities, marked hypokalemia was found in all three patients, but hyperglycemia and osteoporosis were present only in case 2. The clinical heterogeneity is probably due to the large span of ACTH levels from 19.8 to 103.0pmol/L. Bilateral adrenalectomy rapidly and effectively halted hypercortisolism in our case series. The surgery has been traditionally considered a safe option to achieve a radical and fast treatment of hypercortisolism. In a meta-analysis of 23 studies, 30-day morbidity and mortality were acceptable (18% and 3%, respectively) (30). Clinical remission of Cushing’s syndrome was greater than 95%. But the downside of bilateral adrenalectomy is also very obvious, including the need for lifelong glucocorticoid and mineralocorticoid replacement therapy, and the risk of adrenal crisis (4.1 to 9.1 per 100 patient-years). In recent guidelines and reviews regarding EAS, bilateral adrenalectomy has been reconsidered and restricted to very severe cases, when steroidogenesis inhibitors are unavailable, ineffective or poorly tolerated (18). The therapeutic effect of steroidogenesis inhibitors on adrenal medulla-originated EAS needs further exploration. When medical treatment fails, bilateral adrenalectomy should be performed as soon as possible. Survival of patients with EAS is dependent on tumor histology and by the severity of hypercortisolemia. Patients with SCLCs and thymic carcinoids seem to have the worst prognosis, while patients with tumors with endocrine differentiation have a better outcome. Adrenal medulla-originated EAS is relatively benign. Just as our case series, when hypercortisolemia is well controlled, the patients will survive with improved health-related quality of life.

In summary, our case series reveals the adrenal medulla to be a site of ectopic ACTH secretion, which is previously ignored. Adrenal medulla-originated EAS makes the differential diagnosis of ACTH-dependent Cushing’s syndrome much more difficult. Control of the hypercortisolism is mandatory for such patients. When steroidogenesis inhibitors are unavailable, response to somatostatin analogues is limited, or the adverse effects are intolerable, bilateral adrenalectomy is the last choice. If hypercortisolemia is promptly and effectively controlled, the prognosis is often favorable.

## Data Availability Statement

The original contributions presented in the study are included in the article/**Supplementary Material**. Further inquiries can be directed to the corresponding authors.

## Ethics Statement

Written informed consent was obtained from the individual(s) for the publication of any potentially identifiable images or data included in this article.

## Author Contributions

YC analyzed the data. She was a major contributor in writing the manuscript. WG made substantial contributions in interpretation of data, and have been involved in revising the manuscript critically for important intellectual content. JL supervised histological examination. JD, JB, JTD, and SZ made substantial contributions to acquisition of data, or analysis. YM and ZL were the superior advisors. They agreed to be accountable for all aspects of the work in ensuring that questions related to the accuracy or integrity of any part of the work are appropriately investigated and resolved. All authors contributed to the article and approved the submitted version.

## Conflict of Interest

The authors declare that the research was conducted in the absence of any commercial or financial relationships that could be construed as a potential conflict of interest.

## Supplementary Material

Supplementary Figure 1 Pituitary MRI images for case1 **(A)**, case 2 **(B)** and case 3 **(C)**. Pituitary contrast-enhanced MRI did not showed signs of pituitary adenoma.

Supplementary Figure 2 Pituitary MRI image for case 2, revealing the presence of a mass in the left nasal cavity and left ethmoidal sinus (arrow).
