# Supplementary figures and images for: Case Report: Three Rare Cases of Ectopic ACTH Syndrome Caused by Adrenal Medullary Hyperplasia
Source: Front Endocrinol (Lausanne). 2021 Jul 1;12:687809. doi: 10.3389/fendo.2021.687809 (PMC8281927; doi:10.3389/fendo.2021.687809)

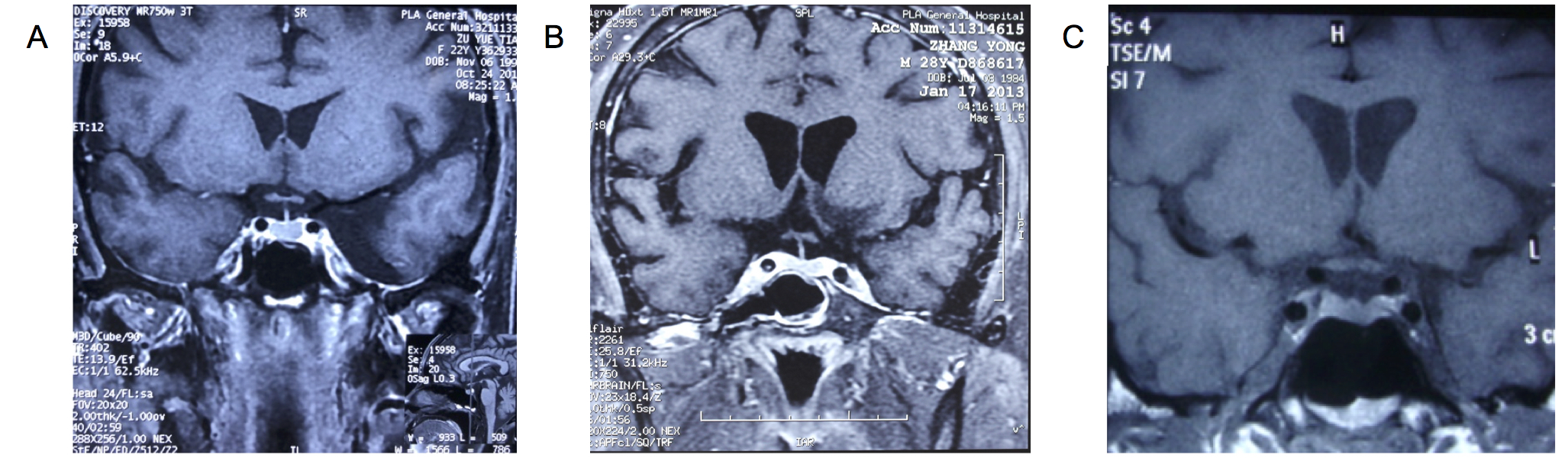

Supplement: Supplementary Figure 1 — Pituitary MRI images for case1 (A), case 2 (B) and case 3 (C). Pituitary contrast-enhanced MRI did not showed signs of pituitary adenoma. [file Image_1.jpeg]

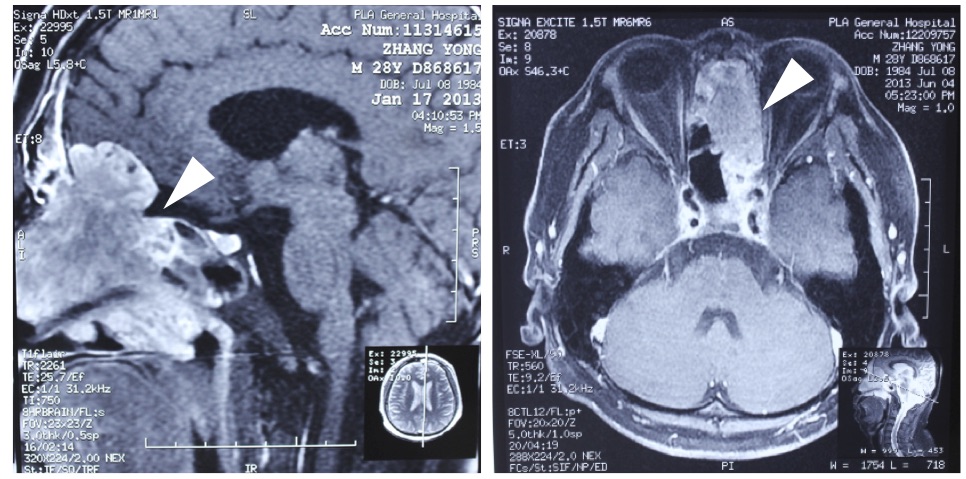

Supplement: Supplementary Figure 2 — Pituitary MRI image for case 2, revealing the presence of a mass in the left nasal cavity and left ethmoidal sinus (arrow). [file Image_2.jpeg]
